# The Role of Mucoadhesion and Mucopenetration in the Immune Response Induced by Polymer-Based Mucosal Adjuvants

**DOI:** 10.3390/polym15071615

**Published:** 2023-03-24

**Authors:** Nathaly Vasquez-Martínez, Daniel Guillen, Silvia Andrea Moreno-Mendieta, Sergio Sanchez, Romina Rodríguez-Sanoja

**Affiliations:** 1 Instituto de Investigaciones Biomédicas, Universidad Nacional Autónoma de México, Circuito, Mario de La Cueva s/n, C.U., Coyoacán, Mexico City 04510, Mexico; vasquezmarnathaly@gmail.com (N.V.-M.);; 2 Programa de Doctorado en Ciencia Bioquímicas, Universidad Nacional Autónoma de México, Circuito de Posgrado, C.U., Coyoacán, Mexico City 04510, Mexico; 3 Consejo Nacional de Ciencia y Tecnología, Benito Juárez, Mexico City 03940, Mexico

**Keywords:** mucoadhesion, polymeric particles, immune response, mucosal vaccines, mucosal adjuvants

## Abstract

Mucus is a viscoelastic gel that acts as a protective barrier for epithelial surfaces. The mucosal vehicles and adjuvants need to pass through the mucus layer to make drugs and vaccine delivery by mucosal routes possible. The mucoadhesion of polymer particle adjuvants significantly increases the contact time between vaccine formulations and the mucosa; then, the particles can penetrate the mucus layer and epithelium to reach mucosa-associated lymphoid tissues. This review presents the key findings that have aided in understanding mucoadhesion and mucopenetration while exploring the influence of physicochemical characteristics on mucus–polymer interactions. We describe polymer-based particles designed with mucoadhesive or mucopenetrating properties and discuss the impact of mucoadhesive polymers on local and systemic immune responses after mucosal immunization. In future research, more attention paid to the design and development of mucosal adjuvants could lead to more effective vaccines.

## 1. Introduction

The development of mucosal vaccines continues to be a priority in the fight against microorganisms whose entry is the mucosa. However, the mucus layer limits the passage of antigens across the epithelium to reach mucosa-inductive sites. This protective barrier facilitates the clearance of foreign pathogens and particles. The mucosal compartments have the epithelium and the mucosal immune system as barriers to defense. The epithelial barrier interconnected by tight junctions, the mucus layer, antimicrobial peptides, and immunoglobulin A (IgA) production prevent, as a whole, access to pathogenic microorganisms, foreign particles, and toxins [1,2,3].

**Figure 1:**
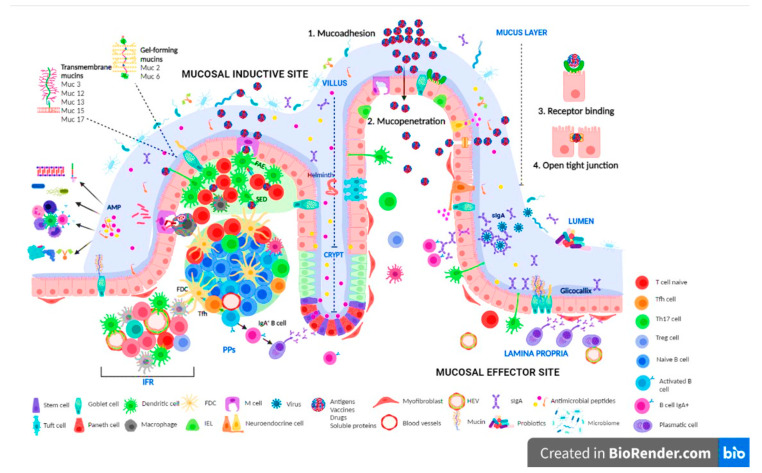
Components of gut-associated lymphoid tissues. The intestinal epithelium comprises multiple cell types derived from intestinal stem cells (IECs), including absorptive enterocytes, Paneth cells, goblet cells, tuft cells, and enteroendocrine cells. The IECs maintain gut homeostasis by synthesizing and secreting mucins, antimicrobial peptides, hormones, and soluble proteins. Furthermore, the IECs participate in the recognition of microorganisms via PPRs. The inner mucus layer or glycocalyx that lines the intestinal epithelium contains many antimicrobial peptides (AMPs) and IgA, both with effector functions. The outer mucus layer is also colonized by commensal microbiota. AMPs bind to glycosylated proteins, neutralize bacterial toxins, participate in the recruitment of effectors cells, and directly kill bacteria. Antigen uptake can occur by M-cell-mediated transcytosis, via macrophage/dendritic cells extending transepithelial dendrites into the gut lumen, paracellular pathway, or through goblet-cells-associated antigen passages. The passage of the particles is further facilitated by mucoadhesion and mucopenetration phenomena. In gut-associated lymphoid tissues, antigens are presented to naive T and B cells with subsequent antigen-specific immune responses. PPs are mucosa-inductive sites for immune responses in the gastrointestinal tract, while the lamina propria and epithelial compartment constitute effector sites. FAE, follicle associated epithelial; SED, subepithelial dome; PPs, Peyer’s patches; IFR, interfollicular regions; FDC, follicular dendritic cells; Tfh, T follicular helper cells.

Furthermore, the mucosal immune system, comprising a network of mucosa-associated lymphoid tissues (MALT), is responsible for initiating and establishing the antigen-specific innate and adaptive immune response following infection or vaccination [4]. Both inducing and effector sites are found in MALT. For example, in the small intestine, immune-inductive gut-associated lymphoid tissue (GALT) comprises the Peyer patches, mesenteric lymph nodes, and isolated lymphoid follicles (Figure 1). The GALT is covered by a follicle-associated epithelium, predominantly composed of enterocytes and membranous cells (M cells) [5]. M cells, surrounded by a thin layer of mucus, are responsible for transferring, via phagocytosis or transcytosis, bacteria and particulate antigens from the luminal side to the basal side of the epithelium and to the subepithelial dome (SED) [6,7], along with the other intestinal epithelial cells [8,9]. Regardless of the sampling mechanism, antigen-specific primed cells in the mucosa leave the encounter site to enter the lymph, then the bloodstream, and re-enter the mucosal tissues of origin, where they differentiate into effector or memory B and T cells, a process mediated by integrins [10]. In the small intestine, this effector site is the lamina propria. For a deeper understanding of the inductive and effector sites in the mucosal immune responses other than GALT and NALT, the following reviews are suggested [11,12,13].

Considering the barrier properties of mucosal surfaces, extensive studies have been performed to develop strategies for prolonging the residence time of vaccines in epithelial tissues; one of the most relevant is the use of mucoadhesive formulations. This review provides an overview of mucus, mucin, and how the interactions between mucus and particles occur. We discuss the physicochemical characteristics of particles that lead to improved mucoadhesion and/or mucopenetration.

The paper aims to show solid scientific evidence to re-evaluate the correlation between improving the mucoadhesive or mucopenetrating characteristics of polymer-based particles used as mucosal vaccine adjuvants and the increase in specific systemic and mucosal immune responses. Likewise, we address potential strategies for mucus penetration, highlighting the importance of incorporating them to design more effective mucosal vaccines. For these purposes, most of the included research papers are those in which the mucoadhesion test of polymer particles was published together with the assessment of adjuvanted capacity following mucosal vaccination.

## 2. Mucosal Vaccination

The mucosal surfaces cover a vast extension of the body surface area. Due to continuous environmental exposure, many pathogens, or antigens, as well as particles found in the air and toxins, have the mucosal surfaces as portals of entry to the body. Therefore, the mucosal tissues play a fundamental role in protecting from invasion by harmful microorganisms through physical and biological barriers. The characteristic induction of antigen-specific sIgA antibodies, both local and distant, as well as protective immunity in systemic and mucosal compartments, makes mucosal vaccination the best tool for reducing mortality and morbidity caused by infectious pathogens that enter the body through the mucosal surfaces [14,15].

However, most vaccines licensed for use in humans are currently administered parenterally. Although parenteral immunization successfully induces a protective systemic immune response, it hardly induces an effective mucosal immune response, and the cellular mechanisms underlying this response remain largely unknown [16,17,18]. 

The SARS-CoV-2 pandemic highlighted mucosal vaccination’s importance in triggering an immune response at the predominant sites of pathogen infections and protecting against mucosal invasion. Several nasal and oral vaccines are currently in the clinical phase (Table 1), thanks to numerous researchers who have focused on developing mucosal vaccination platforms for other diseases in recent decades. It is essential to clarify that there are already authorized vaccines for application through these routes for other diseases. For example, oral vaccines are currently on the market for *Vibrio cholerae* (Dukoral^®^, ShanChol™ OCV, Euvichol-Plus^®^/Euvichol^®^, and Vaxchora^®^) [19], poliovirus (BIOPOLIO™ B1/3), rotavirus (RotaRix^®^, RotaTeq^®^, Rotavac^®^, and RotaSiil^®^) [20], *Salmonella typhi* (Vivotif^®^ Ty21A) [21], and the adenovirus vaccine (Adenovirus types 4 and 7) approved for military use only [22]. On the other hand, presented in the form of a spray for nasal administration, there are the influenza vaccines against type A and B viruses (FluMist^®^ Quadrivalent and Nasovac-S™) [23,24]. Mucosal vaccines induce diverse immune responses in strength, efficiency, and long-term protection [25,26,27]. Most of these formulations contain live attenuated or inactivated whole-cell organisms or viruses [28,29]; consequently, the limitations and challenges are many, especially those related to safety. In this regard, developing subunit vaccines becomes a promissory strategy because they are safer but, unfortunately, less immunogenic. Consequently, most of the time, subunit vaccines demand the use of adjuvants that require specific characteristics for mucosal routes; regrettably, there are no approved vaccines for these routes.

### 2.1. Mucosal Vaccine Adjuvants

The induction of robust immune responses after immunization by mucosal routes requires the antigen on the mucosal surface for its transport across the epithelial barrier. For this reason, the rational design of mucosal vaccines and adjuvants must consider the specific problems related to the route of administration, such as changes in pH, enzymatic degradation, or entrapment in the mucus layer that limit the absorption of antigens. Consequently, it is a challenge to find effective mucosal adjuvants that allow overcoming the limitations associated with the mucosal barrier, enhancing local and systemic immune responses.

Several parenteral adjuvants have been approved for use in humans, including adjuvants based on aluminum hydroxide salts and gels, virosomes, oil-in-water emulsions (MF59^®^, AS03, MontanideTM ISA 51 VG, and ISA 720 VG) [30,31,32], monophosphoryl Lipid A (MPL) [33], Adjuvant Systems^®^ (an immunostimulant combination of AS01 (MPL and QS-21) [34] and AS04 (MPL and aluminum hydroxide)) [35], CpG1018 [36] (a synthetic TLR-9 agonist adjuvant (Dynavax^®^)), and recently, Matrix-MTM (saponins from *Quillaja saponaria*), which were authorized in a recombinant vaccine for SARS-CoV-2 [37]. However, none of these adjuvants have been licensed for human mucosal use, although several mucosal vaccine adjuvants are currently under pre-clinical evaluation [38,39,40]. The rapid progress in SARS-CoV-2 mucosal vaccine development has allowed more adjuvants to be taken to the clinical phase, such as adenoviral vector as self-adjuvant [41], artificial-cell-membrane polymersome-encapsulated CpG (NCT05385991) or membrane vesicles from *Neisseria meningitis* [42] (Table 1). In the pre-clinical phase, N-N-N-trimethyl-chitosan particles designed for mucosal administration are under investigation [43].

#### Use of Particulate Systems as Mucosal Vaccine Adjuvants

**Scheme 1:**
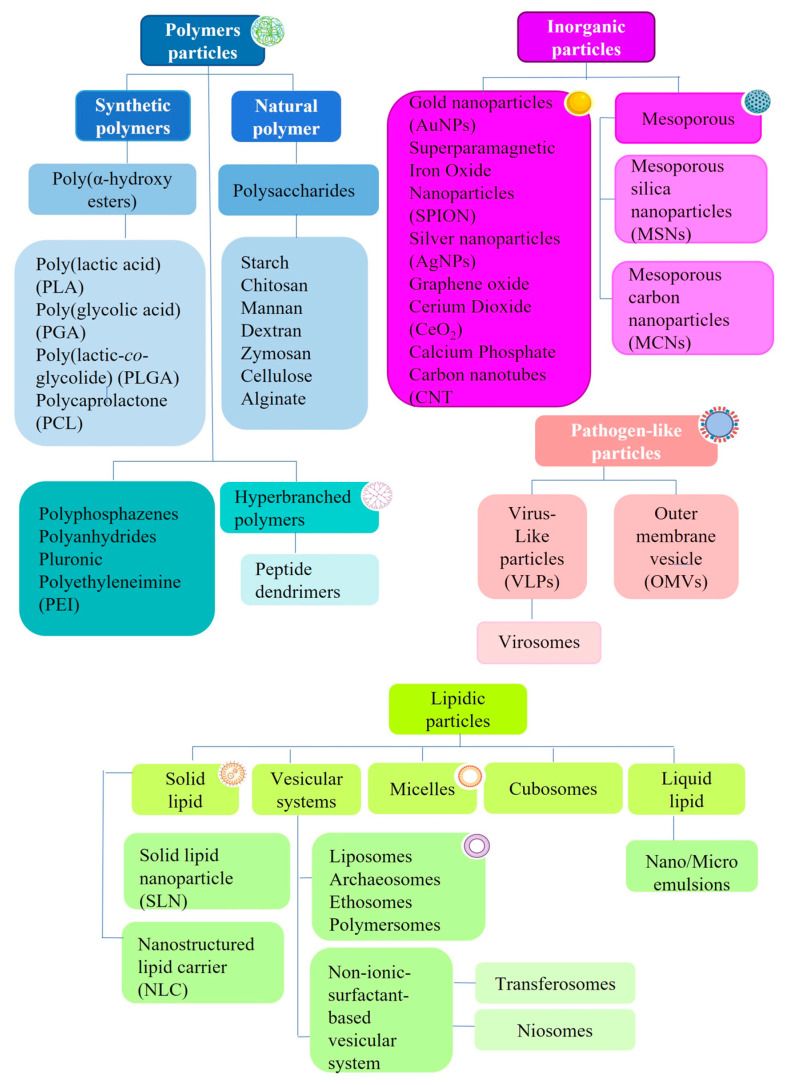
Material types of particulate systems used as mucosal adjuvants. PCL, polycaprolactone; PLGA, poly (lactic-co-glycolide); PGA, poly (α-L-glycolic acid); PLA, poly (lactic acid); γ- PGA, poly (glutamic acid); PEI, polyethyleneimine; VLPs, virus-like particles; MSNs, mesoporous silica nanoparticles; MCNs, mesoporous carbon nanoparticles.

Encapsulation, entrapment, or conjugation of antigens within particulate systems is one of the most promising approaches for the mucosal administration of vaccines. Particles protect the antigens from in vivo enzymatic degradation, prolong the residence time in mucosa favoring delivery and absorption [44], promote the transport of antigens and cells to lymph nodes [45], improve antigen presentation, and enhance immunogenicity [46]. All these benefits are reflected in greater vaccine efficacy [47,48,49,50,51]. Scheme 1 summarizes the general classification of particle-based systems used commonly as vaccine adjuvants.

The performance of these particles as adjuvants is usually determined by their intrinsic characteristics, such as size [52,53,54], shape [55,56], surface charge [57], and hydrophobicity [58,59], and also are influenced by the methods used for antigen loading [60], the density of antigen on the surface [61], the ability of controlled-release kinetics [62], functionalization [63,64,65], and importantly mucoadhesion [66,67]. In many cases, particulate vaccine adjuvants mimic the size, shape, and surface molecule organization of pathogenic microorganisms and can contain molecules such as pathogen-associated molecular patterns, which directly impact the recognition, interaction, phagocytosis, and processing of antigens by antigen-presenting cells (APCs), affecting vaccine efficacy [68,69,70].

## 3. Mucoadhesion

The mucus layer covering the mucosal epithelium is mainly synthesized and secreted by goblet cells [71,72]. Mucus is composed of water (>95%), electrolytes, enzymes, salts, DNA, lipids, growth factors, antimicrobial peptides, immunoglobulins, and mucins, the most abundant high-molecular-weight glycoproteins of the extracellular mucus [73,74]. The mucus layer’s composition, thickness, viscosity, and rheological properties vary widely among mucosal tissues. For instance, the nasal mucus is thinner (10 µm thick), making it highly permeable compared to the mucus layer along the gastrointestinal tract, whose thicknesses range from 180 µm to 800 µm from the antrum to the colon, respectively [75,76]. The mucus’s rheological properties also vary according to the anatomical site and the type, composition, and properties of the mucins [77]; therefore, the transport of microorganisms, molecules, particulate matter, drugs, exogenous, and endogenous agents through the mucus is also different. For a better understanding of essential functions, general features, and distribution according to the anatomical location of mucins, see Table 2.

**Figure 2:**
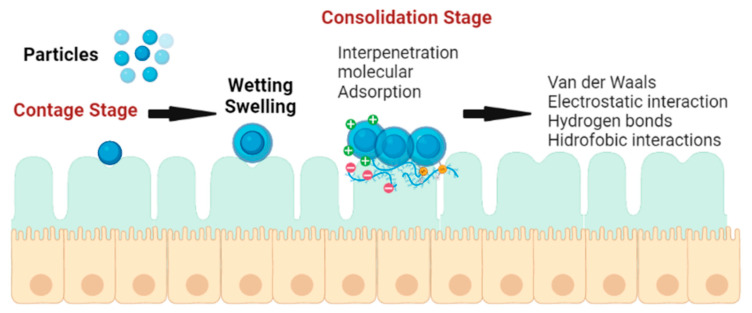
Mucoadhesion of polymeric particles to mucous membranes. First, an intimate interaction between polymeric particles and the mucous membrane occurs in the contact stage, with subsequent wetting and swelling. Later, in the state of consolidation, physicochemical interactions enable the adsorption and molecular interpenetration of polymer chains into the mucus layer that covers the epithelial surface, eventually leading to prolonged adhesion.

Some synthetic and natural materials/macromolecules and hydrocolloids adhere to biological surfaces [78] and remain attached for an extended period of time by interfacial forces. When the adhesive attachment occurs with mucus or epithelial tissues, the phenomenon is considered mucoadhesion (Figure 2) [79] and involves an interaction with mucin.

**Figure 3:**
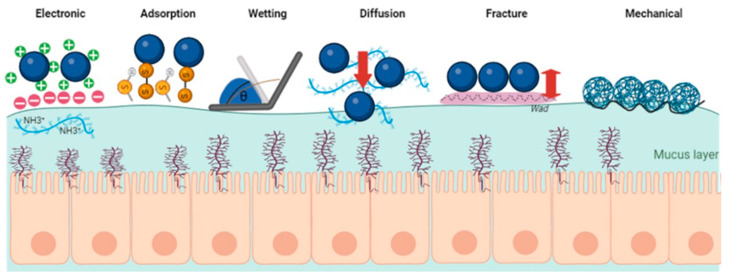
Theories of mucoadhesion. The electronic theory describes mucoadhesion mediated by electrostatic forces. Adsorption assumes intermolecular contact at the interface and adhesion of materials by intermolecular forces. Wetting is described in terms of the spreading coefficient; therefore, the requirement for mucoadhesive materials to adhere is that these can spread spontaneously onto a surface. The diffusion theory: interdiffusion and interpenetration through the polymer-mucin interface according to their concentration gradient. Fracture describes the forces required to separate two surfaces after bonding and assumes that adhesion bond failure occurs at the interface (*Wad*: work adhesion). Mechanical assumes that the irregular rough or abrasive substrate surface provides mechanical keying.

Different theories try to explain the interactions between bioadhesive polymers and mucosal surfaces from both physical and chemical perspectives: the electronic theory [80], the adsorption theory [78], wetting theory [81,82], diffusion theory [83], and fracture and mechanic theory [84] (details in Figure 3). In all of them, the molecules must bind through the interface, an interfacial layer formed between the adhesive and the mucosal tissue. However, the links between the polymers and the mucus differ in each theory.

In addition to pharmacokinetic studies, in vivo and ex vivo methods for assessing mucoadhesion allow for the direct investigation of the particulate systems’ adhesion to mucosal tissues [85,86] or mucosa-mimetic materials [87]. Likewise, it is possible to perform in vitro determinations that depend mainly on the physical properties of the polymers [88]. Several detailed reviews can be consulted to learn more about the methods used in mucoadhesion [89,90,91].

### Determining Factors in the Mucoadhesion and Mucopenetration of Polymeric Particulate Systems

The mucoadhesive properties of particulate adjuvants can be affected by the physicochemical characteristics of polymers, such as size, ζ-potential, elasticity, molecular weight, or spatial conformation, as well as by environmental factors, such as pH and presence of metal ions, and physiological factors, including mucin turnover. The particles are trapped in mucus networks through polyvalent adhesive interactions [92]. Sulfate groups on N-acetylglucosamine and galactose and carboxylic groups on sialic acid sugars confer negative charges to the mucin under most pH conditions [93]. Hydrophilic particles adhere to the negatively charged moieties, while hydrophobic particles are captured by low-affinity bonds between hydrophobic protein residues and particle surfaces. Although cationic polymers have shown better mucoadhesive properties [94], anionic polymers also attach to mucin just as much as cationic or nonionic polymers. This occurs thanks to surface carboxyl groups in mucin that permit interaction via hydrogen bonds with the oligosaccharide chains [95]. Mucus limits the diffusion of particles of any size, so it seems that size has a more significant contribution to mucopenetration than to mucoadhesion. Thus, the transport rate of particles in mucus decreases with increasing particle size [96,97].

The surface functionalization of particles affects adhesion and permeation across the mucus of particles of equal size, i.e., as expected, polystyrene NPs of a small size (100 nm) penetrate the mucus better than large particles (500 nm); however, among 500 nm particles, sulfate-functionalized particles were 1.7 times more permeable than amino-functionalized particles [98].

On the other hand, cylindrical-shaped NPs and rod-shaped nanocrystals have been shown to penetrate the mucus more efficiently compared to their spherical counterparts with similar particle sizes and surface charges [99]. Similarly, compared to their soft and hard counterparts, particles with moderate stiffness have a higher diffusivity through the mucus [100].

## 4. Mucoadhesive and Mucopenetrating Polymer-Based Adjuvants

For at least the past four decades, multiple research groups have searched for materials for the development of mucoadhesive and/or mucopenetrating pharmaceutical formulations to improve the bioavailability of active constituents [101,102,103,104]. Both the adhesive and mucopenetrating properties of particulate adjuvants allow them to reach the epithelial barrier. Once there, the particulate can be transported to the basolateral side to initiate mucosal immune responses.

Several materials commonly used in the pharmaceutical industry are also used as mucosal vaccine adjuvants; however, few studies have been devoted to evaluating the direct influence of mucoadhesion and mucopenetration on the strength and quality of the antigen-specific immune responses stimulated after mucosal vaccination. Consequently, we provide an overview of polymer-based particles in the following sections. Based on the available experimental findings, we analyzed the association between increased mucoadhesive strength, mucosal penetrability, and enhanced immune response quality after mucosal vaccination.

### 4.1. Chitosan and Chitosan Derivatives

Chitosan (CS) is a natural cationic polysaccharide obtained by the deacetylation of chitin. CS has been widely used in various biomedical applications due to its biodegradability, biocompatibility, low toxicity, immunogenicity, and mucoadhesive properties [105,106,107]. The mucoadhesive properties of CS are attributed to the protonation of the amino groups in weakly acidic media, which interact with the negatively charged sialic acid moieties of mucin. However, different chemical processes have been applied to CS to improve its application limitations, such as high hydrophilicity, low solubility from pH 7.4, high degree of swelling, and thermal stability [108,109]. These modifications, in turn, are favorable to promoting adhesion to mucosal surfaces and, as a result, enhance mucosal contact time.

For example, the chemical conjugation of CS with hydrophilic ethylene glycol branches improves solubility in water at neutral and acid pH values and its steric stability [110,111]. Pawar and Jaganathan (2016) compared the immunogenicity of CS NPs and glycol chitosan (GC) NPs loaded with a hepatitis B surface antigen (HBsAg) after nasal administration in Balb/c mice. While the anti-HBsAg antibody titer induced by HBsAg alone was minimal, HBsAg conjugated with GC NPs significantly increased serum IgG and IgA antibody titers in nasal, saliva, and vaginal secretions, compared to the CS-conjugated group. Splenocytes isolated from mice immunized with GC NPs and CS NPs secreted significantly higher amounts of IL-2 and IFN-γ than the control mice immunized with Alum-adsorbed HBsAg. Nasal clearance studies of radiolabeled particles in rabbits showed a nasal cavity retention time of up to 240 min for GC NPs (20% radioactivity) compared to 180 min for CS NPs (20% radioactivity) and 90 min for HBsAg alone (<20% radioactivity). In confirming nasal deposition after nasal administration in mice, only formulations with GC and CS NPs were retained in the NALT at 30 min, with higher fluorescence intensity for GC NPs than FITC-BSA [112].

Similarly, adding cross-linking agents, such as tripolyphosphate (TPP), improves the encapsulation efficiency during the elaboration of CS NPs [113]. Co-crosslinked vanillin/TPP was used for developing a trivalent oral vaccine (DwPT). Studies of the adhesion behavior of the microspheres were related to the ζ-potential of the groups, the electrostatic interaction between the positively charged CS and the negatively charged sialic acid of mucin, and the degree of cross-linking. Thus, the highest swelling index was for the group with the lowest degree of cross-linking. Batches with electropositive charge (placebo CS microspheres, diphtheria toxoid (DT) CS microspheres, and tetanus toxoid (TT) CS microspheres: ~+30 mV) showed a higher adhesion to mucin than those with ζ-potential around +10 mV (whole-cell pertussis (wP) CS microsphere and trivalent (DwPT)). Antibody response in serum corresponded to the mucoadhesion of the microspheres, developing a higher IgG antibody titer in TT and DT batches on days 28 and 35 after immunization, followed by batches with a lower adherence (PT: pertussis toxin). This response was consistent with that observed in saliva and intestinal secretions [114].

Other derivatives of CS have been developed to improve, specifically, absorption and bioadhesion properties. Currently, the most used are obtained by quaternization, acylation, thiolation, and carboxymethylation [109]. Trimethyl chitosan (TMC), a quaternized derivative of CS with polyampholytic properties, improves CS solubility without affecting its mucoadhesive cationic nature, reduces cytotoxicity, and enhances absorption on mucosal surfaces in a wide range of pH values, increasing the carrying capacity [115,116,117].

In 2010, Vyas laboratory used PLGA microparticles (MPs) coated with CS and TMC for the intranasal administration of HBsAg to mice. While unmodified PLGA MPs had a negative ζ-potential (−14.4 ± 1.2), the coating with CS and TMC increased the ζ-potential to values between +5 mV and +10 mV for PLGA/CS MP and +10 mV and +20 mV for PLGA/TMC MP. The authors also indicated that the ζ-potential directly influenced the adsorption capacity of MPs to mucin, i.e., PLGA MPs showed insignificant mucin retention, while CS-PLGA and TMC-coated MPs had significantly higher mucoadhesive properties. Remarkably, this increase in mucoadhesion improved the immunogenicity of the formulation. However, PLGA/TMC MPs induced substantially higher antibody IgG titers throughout the study than PLGA/CS MPs, both in serum and distal mucosal sites [118]. A second study found the same results with PLGA/TMC NPs and demonstrated the adjuvanticity effect of TMC through the stimulation of dendritic cell maturation. Furthermore, TMC-coated MPs were selectively taken up by M cells in the NALT following nasal administration compared to the FITC-BSA solution, which would substantially explain the enhancement of vaccine formulations’ immunogenicity [119].

Another quaternized CS derivative is N-[(2-hydroxyl-3-trimethyl ammonium) propyl] CS or HTCC. HTCC polymers have different degrees of quaternization or extent of positive charge [116]. Zhang et al. prepared OVA-loaded curdlan sulfate-O-HTCC NPs as an intranasal vaccine system. Although the inclusion of curdlan, a β-glucan capable of activating innate immune cells via Dectin-1 receptors and TLR-4 [120], could promote the antigen-specific immune response, its negative surface charge was considered a limitation for mucosal application. For this reason, O-HTCC was added, which, in addition to conferring a positive ζ-potential on the particle, improved its adhesion and subsequent internalization by epithelial cells due to its high viscosity. The OVA–curdlan–O-HTCC complex led to higher OVA-specific CD4+ T-cell, CD8+ T-cell, and B-cell proliferation when nasally administered to mice, compared with the proliferation induced by OVA, OVA–curdlan, OVA–CS, or CS–curdlan [121].

Carboxymethyl chitosan (CMCS) is another water-soluble CS derivative with an improved degradation rate, a desired characteristic for its use in vivo [122]. Recently, CMCS was also used to coat the surface of calcium phosphate (CaP) NPs. The electrostatic interactions and hydrogen bonds between mucin and CaP–CMCS–BSA allowed in vitro adhesion close to 90% compared to CaP–BSA adhesion (60%). Additionally, the diffusion efficiency was higher for CaP–CMCS–BSA than for CaP–CS–BSA, CaP–BSA, and BSA alone. The coating with CMCS and CS improved the apparent permeability coefficient in the mucus layer at 2 h, an index of apical to basolateral transport. Ex vivo biodistribution in a rat study showed that CaP–CMCS–BSA/FITC absorption was improved in the small intestine at 2 h compared to CaP–CS–BSA/FITC, attributed to the change in surface charge caused by coating with CS and its derivative (CaP–CMCS–BSA, ζ-potential: −4.7 mV vs. CaP–CS–BSA, ζ-potential: 8.5 mV). These findings are correlated with the efficacy of oral vaccination since the levels of IgG and sIgA antibodies in sera and feces, respectively, increased after each boost in the animals that received CaP–CMCS–OVA compared to OVA alone [123].

For their part, methyl CS has been studied for diverse biological activities, including as tissue regeneration activator, absorption enhancer, and mucoadhesive [124]. Suksamran et al. evaluated methylated CS MPs for entrapping OVA. Calcium alginate MPs–OVA, calcium alginate–yam starch microparticles (YMP)–OVA, and (YMP)–OVA coated with methylated N-(4-N, N-dimethylaminocinnamyl) CS (TM_65_CM_50_CS) were used in this work. The evaluation of swelling showed that the degree and rate of swelling of the TM_65_CM_50_CS-coated MPs were higher than those uncoated, both in HCL pH 1.2 and in PBS pH 7.4. Similarly, the in vitro mucoadhesion study using the everted gut sac with porcine jejunum showed that, while the adherence percentages of calcium alginate MPs and YMP MPs were low (29.62% and 11.29%, respectively), the coating with TM_65_CM_50_CS of both particles increased mucosal adhesion during the first hour (45.64% and 43.38%, respectively). Oral immunization resulted in significantly higher IgG and IgA levels in mice receiving OVA-loaded TM_65_CM_50_CS-coated MPs, which again confirms the role of mucoadhesive polymers in immunogenicity [125].

The ζ-potential of the CS-based vaccines significantly influences the induction of an immune response affecting more than one mechanism. Jesus et al. demonstrated that. after the intranasal administration of polycaprolactone/CS (PCL/CS) NPs in C57BL/6 mice, the lowest dose of adsorbed antigen (1.5 μg HBsAg) induced antibody titers comparable to the dose containing six times more adsorbed antigen (10 μg HBsAg). Furthermore, this group had the highest number of responding animals. However, serum IgG titers were significantly low compared to previous studies with the same dose of antigen (1.5 μg HBsAg), so the authors suggested that the decrease in ζ-potential (CS: +30 mV) to values close to neutrality generated by antigen interaction (PCL/CS: +26 mV; PCL/CS: 1.5 μg HBsAg: +22 mV; PCL/CS:10 μg HBsAg: +5.7 mV) leads to a reduced uptake in the epithelial barrier. These observations were independent of the mucoadhesive behavior of the particles without antigen evaluated in vitro. Therefore, the authors suggested that the antigen on the particle’s surface reduces the ζ-potential and hinders the interaction with mucin in vivo, avoiding particle–cell interactions and ultimately impacting the immune response [126]. Although this finding contradicts what was observed for other CS-based particles reviewed, it highlights the importance of assessing the mucoadhesion of the polymeric system alone, as well as the particle-entrapped antigens of interest.

### 4.2. Cellulose Derivatives

Carboxymethylcellulose (CMC), an anionic and water-soluble cellulose derivative [127], has been successfully used as a mucoadhesive polymer to enhance immune responses. Hanson et al. developed CMC and alginate (ALG) wafers loaded with the HIV gp140 protein and with α-GalSer as an adjuvant. In ex vivo tests with porcine sublingual mucosa, wafers with a higher CMC content withstood intense mucosal washings and had a higher tissue penetration of the coupled protein (fluorescently labeled bovine serum albumin (BSA)) compared to wafers with a higher ALG content and the free protein. However, the presence of ALG in the formulation was necessary to maintain protein stability on the wafer. Following sublingual administration in mice, most mucoadhesive wafers generated a greater T-cell response in the lungs and cervical lymph nodes [128]. In other studies, it has been suggested that CMC’s viscosity and anionic structure allows the formation of ionic bonding and hydrogen bonds with mucin layers [129,130,131].

### 4.3. Mannan-Decorated Polymeric Particles

Similar results have also been achieved using the dual immunostimulant and mucoadhesive capacity of mannan isolated from the cell wall of *Saccharomyces cerevisiae* [132]. Mannans present immunostimulatory activity via pathogen recognition receptors (PRRs) in APCs. An in vivo optical imaging system, following the intranasal administration of thiolated hydroxypropylmethylcellulose phthalate microspheres (Man-THM), showed that mannan decoration increased the residence time of Cy5.5-conjugated OVA-loaded Man-THM in the respiratory mucosa compared to OVA alone or OVA-loaded THM. Subsequently, the mucosal immune response was evaluated following the nasal immunization of the ApxIIA toxin from *Actinobacillus pleuropneumoniae* loaded in the MPs groups. The findings also demonstrated that the microspheres reached the lungs and secondary lymphoid tissues and induced systemic IgG and secretory IgA responses to the ApxIIA in bronchoalveolar lavage (BAL) and nasal and vaginal washes. Although the immunostimulatory role of mannosylation in enhancing immunogenicity has been reported [133,134], in this work, the authors highlighted the mucoadhesion of the mannosylated microspheres to explain the improved immunogenicity in vivo.

### 4.4. Alginate Coating

Vyas and his team (2014) assessed the coating of CS MPs with alginate (A-CSMp). In contrast to most of the works reviewed up to this point, where the positive surface charge plays a fundamental role in adhesion to mucin, alginate as an anionic polyelectrolyte changes the ζ-potential of the particle to an electronegative value (−29.7 mV). FITC-BSA was rapidly washed from rat jejunal tissues; however, the in vitro retention time in the mucosa was prolonged when FITC-BSA was associated with A-CSMp. In the same way, in the in vivo assays, only A-CSM loaded with FITC-BSA successfully generated uptake by M cells in Peyer’s patches. When evaluating the efficacy of the particulate system in an oral anthrax vaccination model, high-titer anti-PA serum IgA and IgG antibodies were observed in animals receiving particles loaded with antigens compared to the free *Bacillus anthracis* protective antigen [135].

Similarly, Saraf et al. loaded alginate-coated CS NPs (ACNPs) with HBsAg anchored to E. coli EH-100 lipopolysaccharide (LPS) (LPS-HBs-ACNPs) as an adjuvant for oral administration. As expected, the alginate coating changed the ζ-potential of the NPs from +45.2 mV (0.5% CS-0.1% TPP) to −26.2 mV (0.5% CS-0.1% TPP-2% alginate-2% LPS) due to the negatively charged -COO- electrostatic interaction of the alginate on the positively charged -NH3 of the CS. Despite the ζ-potential’s more negative values, in vitro mucoadhesion studies showed that alginate-coated NPs were more mucoadhesive than CS NPs alone. Although anti-HBsAg serum IgG titers were higher for HB-ACNPs after oral administration, sIgA antibody titers in mucosal secretions were higher for LPS-HBs-ACNPs. The anchoring of LPS targeted the NPs to M cells, conferring immunogenicity to the system [136] independently of the mucoadhesive properties of ACNPs. As in the case of LPS, any ligand can be anchored to the particulate system to target it and to allow specific binding to M cells or mucosal epithelial cells. Excellent reviews have been conducted on this topic [137,138,139].

On the other hand, sodium alginate protects the NPs from the hostile environment of the gastrointestinal tract, the same as the introduction of hydrophilic groups, such as hydroxyalkyls, carboxyalkyls, succinyls, and thiols, or polymer grafts, such as PEG. In this way, Amin and Boateng (2022) proposed a system based on OVA-loaded CS NPs coated with sodium alginate or PEG for oral vaccine administration. Both sodium alginate and PEG coatings increased the stability of NPs upon exposure to gastric fluids with the protection of the encapsulated protein (4 h and 1 h, respectively), compared to uncoated NPs (<30 min). After transfer into simulated intestinal fluid, both coatings showed stability for 120 h, although with different release profiles of OVA. Increased alginate concentrations were related to a higher level of mucin binding. According to the authors, the alginate coating ensures stability, allows a higher antigen load to reach the site for mucosal immune response, improves mucoadhesive properties, and enhances the sustained release of antigen-loaded NPs [140].

### 4.5. Xyloglucan

Xyloglucan (XG), a non-anionic polysaccharide and the main hemicellulose component, has been applied with *Quillaja* saponins to vaccine formulations against brucellosis. While *Brucella* LPS was weakly immunogenic, when *B. abortus* LPS-loaded XG NPs were administered nasally to Balb/c mice, higher systemic and mucosal IgG antibody levels and mucosal IgA were induced. Increased immunogenicity was associated with a greater mucoadhesion force of the XG and the LPS-XG NPs compared to the LPS alone, as well as the ex vivo retention of LPS-XG NPs over 24 h in goat mucosa [141].

As in the case of CS, XG has been previously used in pharmaceutical applications in different formulations and by different routes, including mucosal, transdermal, and intraperitoneal, due to its biodegradability, cost-effectiveness, and non-toxicity. Some authors have suggested that the XG molecular structure, “mucin-like,” is responsible for mucoadhesive properties, including swelling capacity and increasing concentration-dependent viscosity [142,143]. All these characteristics, taken together, expand the possibility of the future use of XG in mucosal vaccinations [144].

### 4.6. Poly (Acrylic Acid) and its Derivatives

Poly (acrylic acid) and its derivatives have excellent mucoadhesive capacity compared to cellulose, polycarbophil, chitosan, and pectin [145,146,147]. An example is Carbopol^®^, a highly cross-linked hydrophilic polymer, which provides it with mucoadhesive and viscoelastic properties. Coucke et al. used spray-dried powders of amylopectin (Amioca^®^) with polyacrylic acid (Carbopol^®^ 974P) in different proportions (SD 0/100, 25/75, 50/50, 85/15, and 100/0) for the intranasal administration of H3N2-inactivated influenza virus and in combination with the LTR192G adjuvant in rabbits. The formulation SD25/75 induced the highest serum response of IgG anti-haemagglutinin compared to the formulation SD100/0, thus highlighting the importance of polyacrylic acid. Despite this, neither SD25/75 nor SD0/100 induce a local mucosal response. The immune response was directly related to the negative ζ-potential of Carbopol^®^ 974P and the mucoadhesive properties of the formulations. The reticulated, predominantly elastic, or highly structured characteristics of SD25/75 (G’ >> G’’) increased the residence time in the nasal cavity. In contrast, the lowest viscosity and cross-linking of SD100/0 were associated with a low mucosal retention [148].

### 4.7. γ-PGA

The poly-γ-glutamic acid (γ-PGA)-based vaccine adjuvant, an anionic biopolymer, was used for the intranasal delivery of the influenza fusion protein sM2HA2 and OVA, co-administered with 3-O-desacyl-4′-monophosphoryl lipid A (MPL) and QS21 in a system denominated γ-PGA/MPL/QS21(CA-PMQ). Using in vivo single-positron-emission computed tomography imaging, it was possible to determine that γ-PGA increased the OVA residence time by up to 12 h in the nasal cavity. This signal decreased at 6 h when OVA was administered alone. This result is correlated with the higher serum IgG, IgG1, and IgG2a antibody responses in the groups vaccinated with OVA/CA-PMQ and sM2HA2/CA-PMQ compared to the groups that received OVA and sM2HA2 alone, as well as being superior to that induced by the cholera toxin used as a mucosal adjuvant. Likewise, animals vaccinated with the antigen/CA-PMQ induced more IL-4 and IFN-γ–secreting cell populations in the spleens stimulated with OVA, sM2, and HA2 protein than mice immunized with proteins alone or the control group. Additionally, the CA-PMQ induced high titers of sM2HA2-specific IgA antibodies at the administration and distal sites, along with an increased survival time (80–100%) following the challenge with influenza A subtypes and cleared pulmonary viral titers [149]. The presence of carboxyl groups within γ-PGA can provide a strong interaction with the mucus layer.

The anionic model (−35.5 mV) of Kurosaki et al. with benzalkonium chloride (BK) and γ-PGA NPs in a complex with OVA (OVA/BK/γ-PGA) was used for pulmonary administration. They observed an increased fluorescence intensity in the lung (Alexa647-OVA/BK/γ-PGA) indicative of lung deposition compared to Alexa647-OVA. OVA/BK/γ-PGA increased the levels of specific IgG antibodies, while in the animals that received OVA or the vehicle (BK/γ-PGA), anti-OVA IgG was not detected. The induction of immune responses at the mucosal site was also significantly higher in the OVA/BK/γ-PGA group [150]. Their study did not discuss the role of γ-PGA mucoadhesion in the results obtained. However, the authors suggest the uptake efficiency of BK/γ-PGA NPs by the antigen-presenting cells in the alveolar region. Due to the high capture efficiency of particles <2 μm in the lung [151], the adhesion phenomenon could favor the increased particle residence time in the lung mucosa. Evaluating bioadhesive properties in these systems could help to improve rational vaccine design using polymeric particles.

### 4.8. Thiolated Polymers

The previously reviewed polymer-based adjuvants could be thiolated to improve mucoadhesion. In the past two decades, important research has been conducted using thiolated polymers or so-call “thiomers”, mainly in excipients for drug delivery. Thiomers can interact with mucin through disulfide bonds with the cysteine-rich subdomains of mucus glycoproteins [152]. These covalent bonds are supposed to have stronger binding than the non-covalent interactions that are formed between the polymers and the sialic acid of the mucus layer [153], improving the mucoadhesive properties of the polymers.

Using a tensile test and rotating cylinder method to obtain compressed tablets, Roldo et al. demonstrated that increasing the number of thiol groups covalently attached to chitosan-4-thio-butyl-amidine conjugated significantly improves mucoadhesion compared to unmodified CS. Thiolation increased the total adhesion work (TWA, μJ) up to 100 times [154]. Similarly, thiol reactivity impacts mucopenetration. When the thiol reactivity is medium to low, extensive interpenetration occurs in the mucus layer, with a larger interface for disulfide bond formation. Conversely, highly reactive thiols have difficulty penetrating through the mucus because they form disulfide bonds with the mucins on the surface of the mucus layer, facilitating their rapid removal through mucus turnover [155].

In a recent study, Sinani et al. immunized Balb/c mice with BSA-loaded NPs prepared using aminated CS (aCS) and aminated and thiolated CS (atCS) polymers; mice were nasally immunized at 14-day intervals. At the end of the experiment (day 253), the nanoparticles (aCS and atCS) induced a more robust systemic response, resulting in an almost two orders of magnitude higher systemic IgG titer than the BSA/CpG ODN control, with atCS being the best. These results are correlated with the increased mucoadhesion observed in the aCS and the atCS. Both aCS and atCS modulated the Th2 immune response and enabled immune response at distal mucosal sites [156].

Cellulose acetate phthalate (CAP) is widely used as an enteric coating for pharmaceutical dosage forms due to its solubility at pH values above 6 (such as in the intestines) but poor water solubility at a low pH (such as in the stomach). After exposure to intestinal fluids, the polymer swells, with the subsequent softening or complete dissolution of the phthalate, allowing the release of the biologically active compounds [157]. Lee et al. orally immunized mice with M5BT, a chimerical multi-epitope recombinant protein of foot-and-mouth disease virus (FMDV), alone, loaded in thiolated CAP MPs (T-CAP), or loaded in non-thiolated MPs (CAP). In ex vivo studies in the porcine intestinal mucosa, T-CAP mucoadhesion was 1.48-fold higher than CAP. The improvement in the mucoadhesion properties was reflected in the highest production of antigen-specific IgG antibodies in animals that received M5BT/T CAP. Similarly, this group of animals had significantly higher levels of anti-M5BT IgA in fecal samples at 2 and 4 weeks due to the longer transit time of antigens in the mucosa and increased MHC class II- expression on APC in PPs, related to IgA production [158].

For cationic thiomers such as atCS, the interactions are predominantly driven by electrostatic forces. In contrast, for anionic thiomers, such as T-CAP, interaction with the mucus occurs through hydrogen bonds, van der Waals interactions, and chain entanglement. In both cases, the bioavailability is improved by the extension of the residence time [159]. Notably, regardless of the surface charge of the polymer particles and resulting surface forces, the thiolation of both polymers improved in vivo immune response.

Further evidence has shown that thiomers are susceptible to thiol oxidation at pH ≥ 5, with their effectiveness being reduced following oral administration. Typically, thiol groups (R-SH) can form disulfide bonds with mercaptopyridine substructures, whereby thiol groups are stabilized against oxidation and increase their reactivity. S-protected thiomers, so-called “pre-activated”, have shown greater mucoadhesion than unprotected thiomers, according to Iqbal et al. (2012) [153]. In this work, Iqbal et al. synthesized a polymer with improved mucoadhesive, cohesive, and in situ gelling properties. For this purpose, poly (acrylic acid) (PAA), PAA-cysteine (PAA-cys), and 2-mercaptonicotinic acid (2MNA) coupled with PAA-cys (PAA-cys-2MNA) were compressed into tablets, and the mucoadhesion strength was determined by the rotating cylinder method. Adding thiol groups improved the mucoadhesive properties 456-fold, while the S-protected thiomer increased the contact time to 960-fold compared to unmodified PAA. These thiolated nanosized carriers and others, such as thiolated cyclodextrins [155,160], are research fields that may be explored further for mucosal vaccine development.

## 5. Enhancement of Epithelial Permeability by Polymer-Based Adjuvants

Although mucoadhesive molecules improve the bioavailability of drugs and antigens administered via the mucosa, the mucus layer still limits passage into the epithelium. The transit time of particles in the mucosa is determined by the physiological renewal time of the secreted mucus layer [161]. Mucus turnover reduces the mucosal residence time of particulate delivery systems because they can be trapped by the mucus and rapidly eliminated [162], which could compromise their effectiveness as mucosal adjuvants.

Therefore, polymer-based adjuvants are expected to adhere to the mucous layer, penetrate the epithelium, and reach the inductive sites for mucosal immune responses before being removed. Hence, this section briefly describes the strategies to facilitate mucus barrier penetration and improve the permeability of polymer-based adjuvants once they are in the mucosa.

### 5.1. Mucus-Penetrating Particles

Particles with a low adhesion and small size, thus with few steric hindrances to the mucin network, are often referred to as mucus-penetrating particles. Unlike mucoadhesive particles, mucus-penetrating particles seek to minimize the strength of electrostatic and hydrophobic interactions with the mucin. Polymers with neutral or low positive charges are generally included in the design of mucus-penetrating formulations. Several studies have reported the surface coating of particles with PEG. PEG is used as an adhesion promoter acting at the interface to improve adhesion. Hence, PEG chains tethered or grafted are covalently attached at one end on the polymer surface while the other is free, allowing PEG to diffuse from the polymer network to the mucus and enhancing interpenetration [163]. Wang et al. further demonstrated the formation of hydrogen bonds between the ether oxygen atoms of the PEG chain and glycosylated proteins of mucins. Additionally, they reported PEG with a low molecular weight (2 and 10 kDa), near-neutral surface charge (ζ-potential of 2 ± 4 and 1 ± 3 mV, respectively), minimized mucoadhesion by reducing hydrophobic hydrogen bonding, and electrostatic interactions to have better mucus-penetrating properties. The authors even proposed that PEG-covered particles between −10 and −7 mV are within the interval that defines mucoadhesive vs. mucoinert characteristics [164].

Despite its widespread use in over-the-counter drugs and vaccines, recent approaches suggest that PEG is not immunologically inert [165,166,167]. Several authors demonstrated that introducing PEG to mucosal vaccine formulations increases their protective efficacy [168,169]. Similarly, an extensive recent review explained the impact of PEGylation in terms of biodistribution for anticipating safety and efficacy [170]. Therefore, it is essential to study the tolerability and safety profile of PEG, despite being an alternative to increased mucopenetration.

Some works have also raised doubts about coating particles with PEG due to surface modifications that can alter the linked polymers’ physical and biological properties. Bamberger et al. evaluated the effects on APC response after functionalizing spermine NPs with acetylated dextran (Sp-Ac-DEX) through a process called DEXylation and PEGylation. The average particle size was considerably increased by DEXylation, with subsequent aggregation. PEGylation and DEXylation decreased the primary amines and, therefore, the ζ-potential. This was reflected in the 20% reduction in the cell viability of bone-marrow-derived dendritic cells and macrophages treated with DEXylated NPs, whereas PEGylation treatment increased viability by 10–20% compared to unmodified NPs. However, the binding and cellular uptake of surface-modified NPs was lower in PEGylated particles [171].

Other polymers with mucopenetration ability are poloxamers, also known as Pluronic^®^. These block copolymers consist of hydrophilic poly (ethylene oxide) (PEO), and hydrophobic block-poly (propylene oxide) (PPO) ordered in an A-B-A triblock structure: PEO-PPO-PEO [172,173,174]. Díaz et al. demonstrated that the addition of mucoadhesive and thermosensitive poloxamer 407(F127)-based hydrogels to CS microspheres in a formulation for nasal and conjunctival ram immunization improved both local and systemic humoral immune responses against the BLSOmp31 antigen, an outer membrane protein of *Brucella* spp., along with the reduced excretion of *Brucella ovis* [175]. Pastor et al. proposed a Pluronic^®^ (PF127) and Gantrez^®^ AN119 thermally sensitive hydrogel for intranasal vaccine delivery since the hydrogel increases the residence time of the antigens in the nasal epithelium, allowing their penetration into the deep skin layers of the nose thus reaching the submucosa, where they can trigger an immune response [176].

Another type of mucopenetrant includes nanoemulsions (NEs). Di Cola et al. evaluated PEG-coated O/W NEs with emulsified, added CS as a proposal for the nasal administration of drugs or vaccines. They observed that CS-added NE led to a local shrinking of the mucin gel network, forming larger pores between the mucin bundles. This phenomenon does not occur in the absence of CS. The SAXS (small-angle X-ray) monitoring of the penetration of solute CS-added NE into the PGM showed a higher diffusion over time (20 min) through the mucus mesh. SANS (neutron scattering) confirmed that, unlike the steric hindrance caused by the pore-like size of mucus caused by mucoinert NPs, the CS-added NE based on Solutol^®^ mucopenetrates by the collapse of the mucus mesh [177].

Coating dextran particles with mucopenetration properties have also been explored to improve drug administration performance [178,179,180,181] and enhance immunoadjuvant activity in vivo [182]. Other strategies, such as coating polymeric particles with polydopamine (PDA) [183] or cell-penetrating peptides [184] used in drug delivery, might be explored and characterized in mucosal vaccines, as well as continuing the search for new adjuvants with mucopenetrating properties.

### 5.2. Permeation of Polymeric Particles via the Mucus Layer

An additional consideration for the design of polymer-based particles is passing through the second barrier, the epithelial cell membrane. The permeability of peptides, proteins, and drugs is often deficient. In this sense, absorption enhancers have been developed, which, in addition to preventing enzymatic degradation, facilitate the opening of the epithelial barrier and improve absorption through intracellular or paracellular mechanisms [185]. Absorption and permeation enhancers include surfactants, such as bile salts, fatty acids, phospholipids, tight junction modulators, cyclodextrins, and detergents [186,187,188,189]. This group also includes mucolytic agents, such as acetylcysteine or enzymes, which can decrease the elastic properties and dynamic viscosity of the mucus, influencing the integrity of the mucus layer [190]. For example, Zhang et al. reported the oral administration, in mice, of self-assembled nanoparticles with recombinant urease subunit B from *Helicobacter pylori*, coated with a cell-penetrating peptide, and coated with PEG derivative. NPs were transported transepithelially, improving the systemic and mucosal antibody response and the protection against *H. pylori* after the challenge [191]. It will be essential to continue studying absorption enhancers in mucosal vaccine formulations to improve the immune response.

## 6. Challenges and Opportunities

For several decades, many polymer-based particles have shown promise as potential human mucosal vaccine adjuvants due to their biodegradability, biocompatibility, and nontoxicity characteristics. Added to this is the extensive study of the adjuvant mechanisms of particulate systems. However, in the mucosa, the mucin networks that cover the compartments are often considered a barrier for the particles, so the mucoadhesive and mucopenetrating capacity of the polymer-based particles often defines their adjuvant mechanism of action.

The search for polymers with better mucoadhesive properties, regardless of the polymer’s source, but focusing on the physicochemical characterization of polymeric particles and the contribution of these properties to mucoadhesion, will allow the rational design of mucosal vaccines. However, it is not an easy task because, on the one hand, the smallest nanoparticles are the most mucopenetrating. Still, on the other, there is a lack of studies that suggest an ideal surface charge or a hydrophobicity that favors adhesion. At the same time, it cannot be ignored that there are multiple other cellular mechanisms to elicit the immune response triggered by the polymeric particles, i.e., enhanced antigen uptake, immune cell presentation and recruitment, and traffic to lymph nodes.

Studies demonstrating the correlation between the observed immune response, the physicochemical characteristics, the mucoadhesion, and the mucopenetration ability are scarce. More studies that examine all these factors simultaneously are required to position mucoadhesion as another immune response mechanism necessary for designing more efficient polymer-based particulate adjuvants.

## 7. Conclusions 

The COVID-19 pandemic highlighted the need for mucosal vaccination as an effective strategy to eradicate infectious diseases that have the mucosa as a natural route of infection. Mucoadhesion is probably the most important feature to improve local and systemic immune responses since, by prolonging the residence time of particulate polymers in mucosal tissues, the absorption and sometimes penetration through the mucosal epithelia are allowed and improved. In this sense, studying the physicochemical characteristics of the polymeric particles used as mucosal vaccine adjuvants and how they affect mucoadhesion is crucial to developing new mucosal vaccines.

## Figures and Tables

**Table 1:** Current mucosal COVID-19 vaccine candidates in clinical trials. Adapted from WHO vaccine tracker and landscape, February 2023 (Available online: https://www.who.int/publications/m/item/draft-landscape-of-COVID-19-candidate-vaccines, accessed on: 10 February 2023). RBD, receptor-binding domain; Ad, adenovirus; MVA, modified vaccinia Ankara; LAIV, live attenuated influenza virus; NDV, Newcastle disease virus. **R**, route of administration: in, intranasal; or, oral; ae, aerosol; ih, inhale.

Protein Subunit				
Antigen	Name	Developer	Clinical Trial	R
Spike β-variante	ACM-001	ACM Biolabs	Phase 1 NTC05385991	in
RBD of S protein+ AgnHB	CIBG-669	CIGB, Cuba	Phase 1/2 RPCE0000345	in
RBD of S protein	Cov2-OGEN1	USSF/VaxForm	Phase 1 NCT04893512	or
Spike	OMV-linked Hexapro	Intravacc B. V	Phase 1 NCT05604690	In
**Live attenuated virus**				
S protein	Mv-014-212	Meissa Vaccines, Inc.	Phase 1 NCT04798001	in
S protein	COVI -VAC	CODAGENIX Inc./Serum Institute of India	Phase 3 ISRCTN15779782	in
RBD of S protein	Razi Cov Pars	Razi Vaccine and Serum Research Institute	Phase2IRCT20201214049709N2	In
**Viral vector**				
NON-REPLICANT				
▪ *Adenoviral vector*				
S protein	ChAdOx1 nCoV-19	University of Oxford	Phase 1 NCT04816019	in
S + nucleocapside	VXA-CoV-2-1-Ad5	Vaxart	Phase 2 NCT04563702	or
S protein	BBV154	Bharat Biotech International Limited	Phase 3 CTRI/2022/02/040065	in
RBD	Ad5-nCoV	CanSino Biological Inc./Beijing Institute of Biotechnology	Phase 4 NTC05303584	ih
Spike + nucleocapsid	Ad5-triCoVMac	McMaster University	Phase 1 NTC05094609	ae
*PIV5 vector*				
Spike	CVXGA1	CyanVac LLC	Phase 1 NCT04954287	in
▪ *Ankara vector*				
Spike	MVA-SARS-2-ST	Hannover Medical School	Phase 1 NCT05226390	ih
▪ *Influenza vector*				
N protein fragment	Corfluvec	Research Institute of Influenza	Phase 1/2 NCT05696067	In
REPLICANT				
▪ *Intranasal flu-based RBD*				
RBD of S protein	DelNS1-2019-nCoV-RBD-OPT1	University of Hong Kong, Xiamen University, and Bejing Wantai Biological Pharmacy	Phase 3 ChiCTR2000037782	in
▪ *Live recombinant Newcastle-disease-virus-vectored*				
Spike	NDV-HXP-S	Sean Liu, Icahn School of Medicine at Mount Sinai	Phase 2/3 NTC05354024	In
**Others**				
▪ *DNA based vaccine*				
S protein	BacTRL-Spike DNA vaccine	Symvivo Corporation	Phase 1/2 NTC04845191	or
*Bacterial antigen-spore expression vector*				
Spike	*B. subtilis spores*	DreamTec Research Limited	Phase 1 NA	or

**Table 2:** Overview of mucins. The structural and functional details of mucins are described. Mucins (MUC) are differentially expressed in mucosal tissues. PTS domains: proline/threonine/serine-rich domains; VNTRs: variable number of tandem repeats; GalNAc: N-acetylgalactosamine; OH-: hydroxyl groups; S: serine; T: threonine.

MUCINS	
**General characteristics**	▪ High-molecular-weight glycoproteins (10–40 MDa)
	▪ Molecular organization: a central region with PTS domains with a variable number of VNTRs
	▪ Domains: Highly glycosylated via O-glycosidic linkages between GalNAc and the OH- of S and T residues from the protein core
	▪ In the gastric mucus, some of these sugars bear sulfate ester groups
	▪ A high proportion of cysteine domains participate in dimer formation and form multimers
**Classification**	▪ Membrane-bound/transmembrane
	▪ Secreted/gel-forming
	▪ Soluble
**Biological functions**	▪ Prevents the adhesion of pathogens, foreign debris, or cells to the mucosal epithelium by steric hindrance
	▪ Establishes a selective filter for the diffusion of particles and small molecules
	▪ Bound by and enriched with nutrients, allowing them to harbor beneficial microbial communities
	▪ Attenuates the virulence of opportunistic microorganisms
	▪ Regulates signal transduction
**Anatomical location**	**Type**
**Oral cavity**	MUC1, MUC4, MUC5B, MUC7, MUC19
**Eye**	MUC1, MUC4, MUC16, MUC20, MUC21, MUC22. MUC2, MUC5B, MUC5A, MUC5B, MUC5AC, MUC7, MUC19
**Respiratory tract**	MUC1, MUC4, MUC16, MUC20, MUC5AC, MUC 5B, MUC19
**Stomach**	MUC1, MUC5C, MUC6
**Small intestine**	MUC13, MUC17, MUC2
**Colon**	MUC2
**Female reproductive tract**	MUC1, MUC4, MUC5
**Male urogenital tract**	MUC1, MUC19, MUC20, MUC5B, MUC6

## Data Availability

Not applicable.
